# *APOE* ε2-Carriers Are Associated with an Increased Risk of Primary Angle-Closure Glaucoma in Patients of Saudi Origin

**DOI:** 10.3390/ijms25084571

**Published:** 2024-04-22

**Authors:** Altaf A. Kondkar, Taif A. Azad, Tahira Sultan, Tanvir Khatlani, Abdulaziz A. Alshehri, Rakesh Radhakrishnan, Glenn P. Lobo, Ehab Alsirhy, Faisal A. Almobarak, Essam A. Osman, Saleh A. Al-Obeidan

**Affiliations:** 1 Department of Ophthalmology, College of Medicine, King Saud University, Riyadh 11411, Saudi Arabiaealsirhy@ksu.edu.sa (E.A.); salobeidan@ksu.edu.sa (S.A.A.-O.); 2 Glaucoma Research Chair in Ophthalmology, College of Medicine, King Saud University, Riyadh 11411, Saudi Arabia; 3 King Saud University Medical City, King Saud University, Riyadh 11411, Saudi Arabia; 4 Department of Blood and Cancer Research, King Abdullah International Medical Research Center, King Saud Bin Abdulaziz University of Health Sciences, Ministry of National Guard Health Affairs, Riyadh 11426, Saudi Arabia; 5 Department of Ophthalmology, Imam Abdulrahman Alfaisal Hospital, Riyadh 14723, Saudi Arabia; 6 Department of Ophthalmology and Visual Neurosciences, University of Minnesota, Minneapolis, MN 55347, USA; rakeshr@umn.edu (R.R.);

**Keywords:** apolipoprotein E, angle-closure, genetics, glaucoma, polymorphisms, pseudoexfoliation, rs429358, rs7412, Saudi

## Abstract

This study investigated the association between apolipoprotein E (*APOE*) gene polymorphisms (rs429358 and rs7412) and primary angle-closure glaucoma (PACG) and pseudoexfoliation glaucoma (PXG) in a Saudi cohort. Genotyping of 437 DNA samples (251 controls, 92 PACG, 94 PXG) was conducted using PCR-based Sanger sequencing. The results showed no significant differences in the allele and genotype frequencies of rs429358 and rs7412 between the PACG/PXG cases and controls. Haplotype analysis revealed ε3 as predominant, followed by ε4 and ε2 alleles, with no significant variance in PACG/PXG. However, *APOE* genotype analysis indicated a significant association between ε2-carriers and PACG (odds ratio = 4.82, 95% CI 1.52–15.26, *p* = 0.007), whereas no notable association was observed with PXG. Logistic regression confirmed ε2-carriers as a significant predictor for PACG (*p* = 0.008), while age emerged as significant for PXG (*p* < 0.001). These findings suggest a potential role of ε2-carriers in PACG risk within the Saudi cohort. Further validation and larger-scale investigations are essential to elucidate the precise role of *APOE* in PACG pathogenesis and progression.

## 1. Introduction

Glaucoma encompasses a spectrum of multifactorial ocular disorders marked by gradual deterioration of retinal ganglion cells (RGCs), optic nerve damage, and visual field impairment, often culminating in irreversible blindness if left untreated [1]. Among the subtypes of glaucoma, primary angle-closure glaucoma (PACG) and pseudoexfoliation glaucoma (PXG) exhibit distinct etiologies and clinical presentations [2,3]. PACG is characterized by the narrowing or closure of the drainage angle in the eye, often due to a shallow anterior chamber or a forward-positioned lens. This closure leads to increased intraocular pressure (IOP) and consequent optic nerve impairment [2]. On the other hand, PXG represents a form of secondary open-angle glaucoma, marked by the deposition of pseudoexfoliation material within various ocular tissues, such as the lens capsule, trabecular meshwork, and iris. This material obstructs aqueous drainage from the eye, resulting in increased IOP and optic nerve damage [3]. PACG and PXG significantly contribute to visual impairment and blindness globally, highlighting the importance of early detection and timely intervention to preserve vision and enhance patients’ quality of life [4].

Substantial evidence indicates a genetic predisposition to glaucoma, including PACG and PXG [5,6]. Given glaucoma’s complex and heterogeneous nature, elucidating the genetic factors contributing to PACG and PXG holds significant promise for advancing our understanding of their pathogenesis. This understanding is crucial not only for unraveling the pathogenesis but also for identifying early diagnostic markers, therapeutic interventions, and personalized management strategies [5,6,7].

Apolipoprotein E (*APOE*), a polymorphic gene located on chromosome 19q13.2, encodes a protein crucial for lipid metabolism and transport within the central nervous system and various ocular tissues [8,9]. APOE has also been detected in the pseudoexfoliative material [10]. Two common polymorphisms, rs429358 (T>C) at codon 112 and rs7412 (C>T) at codon 158, in the *APOE* gene give rise to three predominant *APOE* alleles in humans: ε2, ε3, and ε4. Each isoform differs subtly in amino acid composition at positions 112 and 158, resulting in distinct functional properties and disease-risk profiles [11]. APOE ε3, the most prevalent isoform in approximately 77% of the population, features a cysteine at position 112 and an arginine at position 158, and is considered neutral. In contrast, APOE ε4, characterized by arginine at both positions, is reported to increase the risk of atherosclerosis [12], Alzheimer’s disease, and other neurodegenerative conditions [13,14]. On the other hand, APOE ε2, with cysteine residues at both critical positions, is linked to a lower risk of Alzheimer’s but a heightened risk of type III hyperlipoproteinemia [15] and age-related macular degeneration [16,17].

Additionally, rare variants like APOE3-R136S (APOE3-Christchurch), APOE3-V236E (APOE3-Jacksonville), and APOE4-R251G [18,19,20] are thought to protect against Alzheimer’s disease. Beyond this, the *APOE* gene has garnered considerable attention in the field of neurodegenerative diseases, including those implicated in glaucomatous optic neuropathy [16,21,22,23]. Investigations exploring the association between the two common *APOE* polymorphisms and glaucoma susceptibility have yielded conflicting findings across different populations, including Saudi Arabia [24,25,26,27,28]. However, the precise role of *APOE* variants in PACG and PXG pathogenesis still needs to be elucidated, particularly within ethnically diverse populations such as those of the Saudi Arabian Peninsula.

Saudi Arabia, characterized by a high prevalence of consanguineous marriages and a distinctive genetic profile, offers a valuable setting for genetic studies on complex diseases such as glaucoma [29]. Investigating the *APOE* genotype distribution and its correlation with PACG and PXG in this population could provide crucial insights into the genetic determinants of these conditions, potentially uncovering novel biomarkers and therapeutic targets.

Based on this background, the present study aims to explore the genetic association of *APOE* polymorphisms (rs429358 and rs7412) in a PACG and PXG cohort of Saudi origin. Through genotyping analysis of these polymorphisms in PACG and PXG patients and ethnically matched controls, we seek to unravel potential associations between *APOE* genetic variants and glaucoma subtypes within this population.

## 2. Results

### 2.1. Demographic Characteristics of Study Cohort

**Figure 1:**
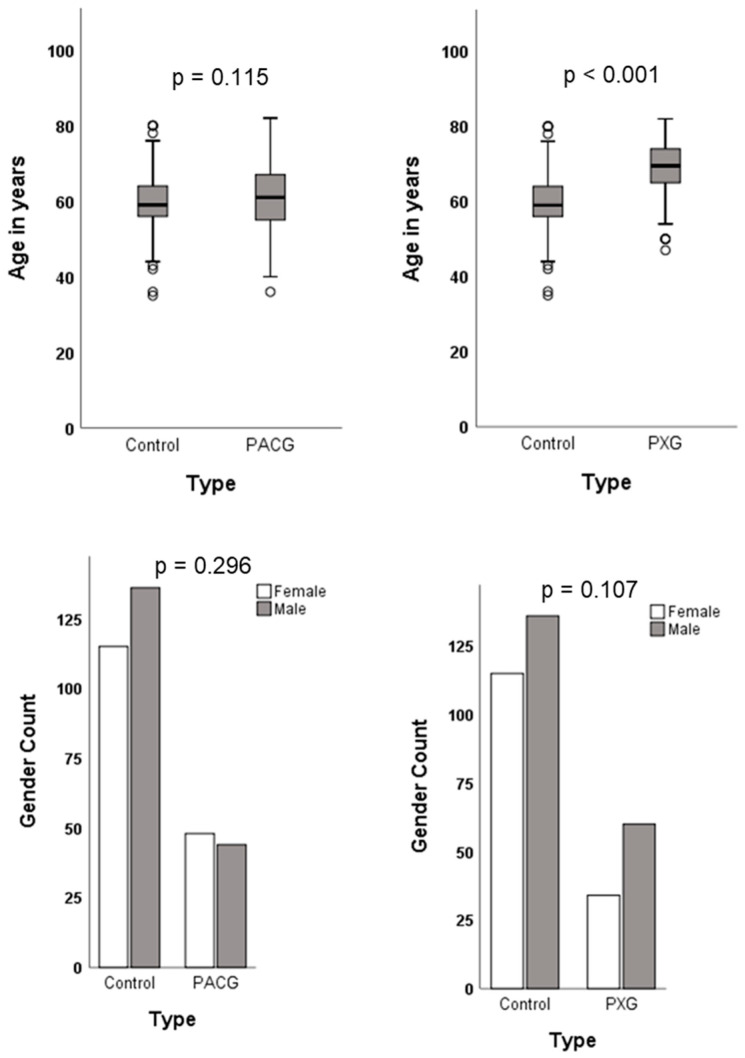
Demographic data of study cohort.

The demographic characteristics of the patient and control groups are illustrated in Figure 1. The mean ages of the study cohort were 59.7 (±7.0) years for the controls, 60.8 (±8.7) years for the PACG patients, and 68.8 (±7.7) years for the PXG patients. In the control group, there were 136 (54%) males and 115 (46%) females, while in the PACG group, there were 44 (48%) males and 48 (52%) females. Among the PXG patients, 60 (64%) were males and 34 (36%) were females. Age and gender distributions did not significantly differ between the PACG patients and controls. However, the PXG patients were significantly older than the controls (*p* < 0.001), with no significant difference in gender distribution.

### 2.2. Association Analysis of rs429358 and rs7412 in the APOE Gene

We analyzed individual polymorphisms rs429358 and rs7412 in the *APOE* gene to determine their association with PACG and PXG. The polymorphisms showed no significant deviation from the Hardy–Weinberg equilibrium [30] (Table 1). The minor allele frequencies (MAF) of rs429358 and rs7412 were 0.10 and 0.03 in the controls, 0.11 and 0.05 in PACG, and 0.06 and 0.04 in PXG, respectively. There was no significant difference in MAF distribution between PACG and PXG compared to the controls (Table 1).

Genotype associations of *APOE* polymorphisms with PACG and PXG were examined using different genetic models. However, none of the polymorphisms showed significant associations (Tables S1 and S2). While rs429358 exhibited a moderately significant association with PACG in the recessive model (*p* = 0.044), this significance did not survive Bonferroni’s correction for multiple testing (0.05/2 = 0.025). Furthermore, this association lost significance after adjusting for age and gender (*p* = 0.063) (Table S1).

### 2.3. APOE Haplotype Association with PACG and PXG

**Figure 2:**
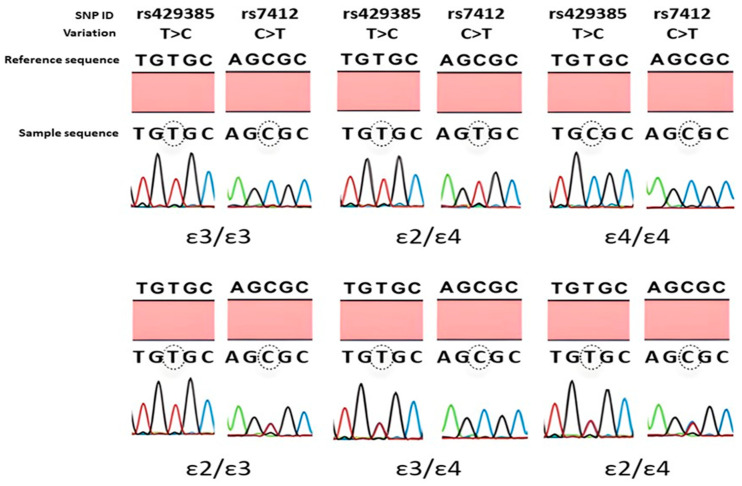
Representative sequencing results of *APOE* genotypes. The genotype calling was based on rs429358 (T>C) and rs7412 (C>T) polymorphisms. The circled nucleotide indicates the position of the nucleotide change compared to the reference sequence. Homozygous ε3/ε3 shows T/T and C/C, ε2/ε2 has T/T and T/T, ε4/ε4 has C/C and C/C, at the circled positions for rs429358 and rs7412, respectively. Likewise, heterozygous ε2/ε3 shows T/T and C/T, ε3/ε4 has T/C and C/C, ε2/ε4 has T/C and C/T, at the circled positions for rs429358 and rs7412, respectively.

The haplotypes of the investigated polymorphisms in the *APOE* gene correspond to different *APOE* alleles (ε3, ε2, ε4) and genotypes (ε3/ε3, ε2/ε2, ε2/ε3, ε2/ε4, ε3/ε4, ε4/ε4). These genotypes were identified depending on the presence of T>C and C>T nucleotides at rs429358 and rs7412, respectively. The representative sequencing results of the identified *APOE* genotypes are presented in Figure 2.

This study explored the haplotype association of *APOE* alleles and genotypes with PACG and PXG, as summarized in Table 2 and Table 3. In the controls, *APOE* ε3 was the most common allele with a frequency of 87.6%, followed by ε4 (9.6%) and ε2 (2.8%). Similar trends were observed in the PACG and PXG patients. None of the allele distributions were significantly associated with PACG and PXG (Table 2 and Table 3). However, the distribution of the six different *APOE* genotypes was significant in PACG (Pearson chi-square = 16.36, df = 5, *p* = 0.006). The ε2/ε3 heterozygotes were found to increase the risk of PACG by over 5-fold, which was statistically significant (*p* = 0.009). Additionally, ε2-carriers had a significant 4.8-fold increased risk of PACG (*p* = 0.007) (Table 2). No significant associations were observed in the PXG patient group (Table 3).

### 2.4. Logistic Regression Analysis of Risk Factors on Glaucoma Outcome

We further investigated the effects of risk factors such as age, gender, and *APOE* genotypes (ε3/ε3, ε2-, ε4-carriers) on the outcome of glaucoma (PACG and PXG) using logistic regression analysis. The analysis revealed statistically significant effects of *APOE* genotypes (*p* = 0.024) and ε2-carriers (*p* = 0.008) in the PACG patients. In the PXG patients, age emerged as a significant predictor (*p* < 0.001), with no significant effect observed for *APOE* genotypes (Table 4). When examining individual *APOE* variants (rs429358 and rs7412), age, and gender in relation to the risk of developing PACG or PXG, none of these variables showed a significant impact, except for age in the PXG patient group (*p* < 0.001). The effects of polymorphism were assessed using both co-dominant and dominant models (Table S3).

### 2.5. Association between APOE Genotypes and Clinical Parameters of Glaucoma

**Figure 3:**
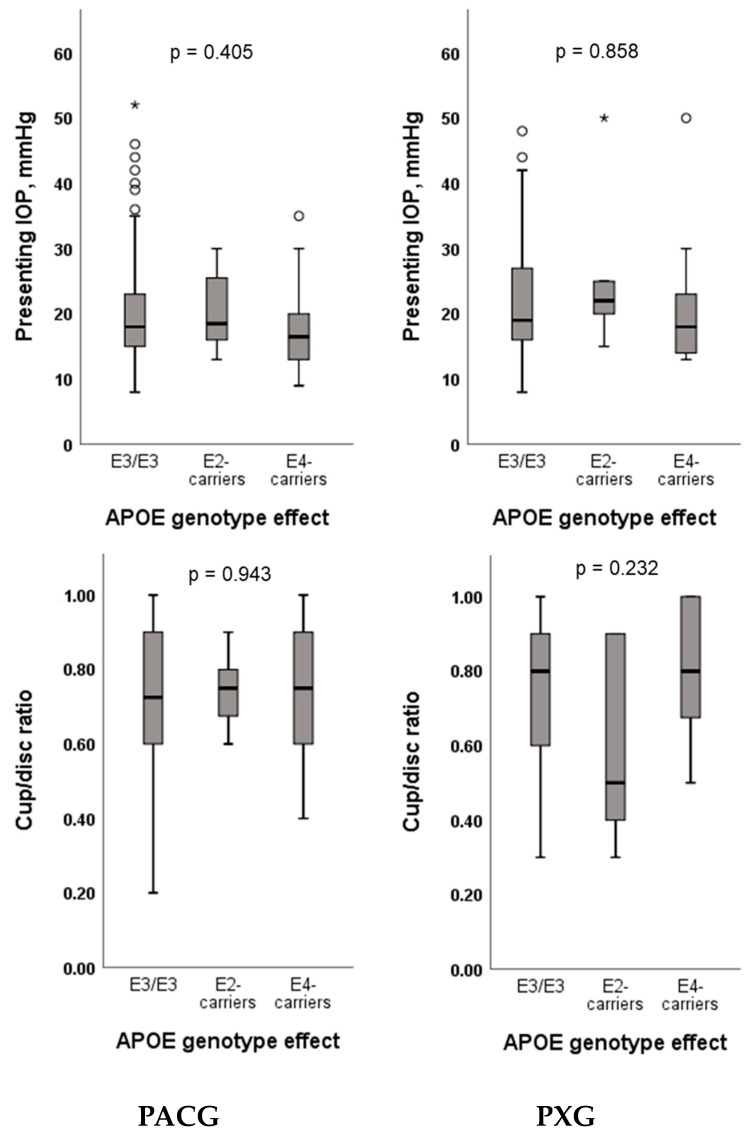
Association analysis of IOP and cup/disc ratio with *APOE* genotypes in primary angle-closure glaucoma (PACG) and pseudoexfoliation glaucoma (PXG) patients. * Outlier.

This study examined whether *APOE* genotypes correlate with clinical parameters of glaucoma, such as IOP and cup/disc ratio in both the PACG and PXG patients. However, no significant effects of *APOE* genotypes (ε3/ε3, ε2-, ε4-carriers) on IOP or cup/disc ratio were observed (Figure 3). Similarly, individual analysis of *APOE* polymorphisms (rs429358 and rs7412) did not show any significant association with IOP or cup/disc ratio (Figures S1 and S2).

## 3. Discussion

The genetic variants of *APOE* (ε2, ε3, and ε4) are associated with the risk of developing several human diseases [31]. Understanding the function and impact of APOE in human health and disease remains a significant focus of research in neurology, cardiology, and ophthalmology [31,32]. The precise influence of genetic factors and polymorphisms in the complex polygenic nature of glaucoma among patients of Saudi Arabian descent remains poorly understood. Herein, we present findings indicating a positive association between the *APOE* ε2-carriers and PACG, but no association in PXG, in a Saudi cohort.

As illustrated in Table 5, the rs429358 and rs7412 polymorphisms in the *APOE* gene responsible for a Cys/Arg interchange and their haplotypes give rise to three major allelic *APOE* variants ε2, ε3, and ε4 [33]. The frequency distribution of these three major alleles varies worldwide (Table 6) [34,35]. While studies on non-human primates suggest the ε4 allele as the ancestral variant [36], modern human populations predominantly exhibit the ε3 variant, with frequencies ranging from 0.968 in Indians to 0.486 in Papuans [34,35]. The ε4 allele ranks as the second most common, with notably high frequencies observed among Pygmy populations in Central Africa (0.407), Khoisan populations in Southern Africa (0.370), Oceanians (including Papuans at 0.368 and Australian Aborigines at 0.260), and the European Saami people (0.310) [34]. In contrast, the ε2 allele is less common, ranging from rare to absent in Native Americans, Siberians, and Mongolians, but relatively more prevalent among Swedish (0.119), sub-Saharan African (0.116), Malay (0.140), and Papuan (0.145) populations [34,35,37]. Our study similarly reflects this global pattern, showing *APOE* ε3 as the most common allele (0.876), followed by ε4 (0.096) and ε2 (0.028). Generally, allele frequencies can vary significantly across populations due to factors such as genetic drift, migration, and natural selection [34,35,38]. Notably, our investigation found no association between the allele frequencies of rs429358, rs7412, and *APOE* haplotype (ε2, ε3, and ε4) and PACG/PXG compared to the controls.

The statistical evidence supporting a causal association between *APOE* variants and glaucoma remains less robust. Numerous studies with inconsistent findings have explored the association between *APOE* alleles/genotypes and adult-onset primary open-angle glaucoma (POAG) in different populations. In Japanese OAG patients, the ε3 allele increased OAG risk, the ε2 allele reduced risk, and the ε4 allele was linked to lower IOP [39]. Conversely, a smaller study in Saudi-origin POAG patients found a significant association with the ε4 allele [27], but our own study in a larger Saudi cohort contradicted this [28]. In Massachusetts and Canadian studies, the ε4 allele showed protective effects in POAG [24,40]. By contrast, in Brazilian POAG cases, the ε2 allele was associated with increased risk [41], while the ε4 allele was linked to neuroretinal thinning in normal-tension glaucoma (NTG) [42]. Conflicting results persist across diverse ethnic groups, including European [43], Chinese [44], Japanese [45], Turkish [46], and in meta-analyses [25,47] reflecting population-specific differences.

On the other hand, few studies have explored the association of *APOE* with PACG and PXG. A study in Saudi PACG patients found no association with *APOE* alleles and genotypes [27], and similar results were reported in large cohorts of German and Italian PXG patients [48]. Another study in Greek patients reported no *APOE* association in pseudoexfoliation syndrome (PXS)/PXG but found an increased risk of POAG in *APOE* ε2-carriers [49]. In a Turkish cohort, *APOE* ε2-carriers were at significantly increased risk of PXS [50]. However, this finding was not replicated in another Turkish study [51]. A recent Finnish study found that the *APOE* ε4 allele protects against POAG and NTG but not against PXG [32].

In our Saudi cohort, *APOE* ε2-carriers were found to be at significantly increased risk of PACG. However, similar to our earlier findings in a POAG cohort [28], no association of *APOE* variants was observed in PXG. A previous study has shown that ε2-carriers had significantly lower IOP than non-ε2-carriers in PXS patients [52]. While ε2-carriers in our study exhibited notably lower cup/disc ratios compared to ε3/ε3 and ε4-carriers in the PXG patients, however, no significant associations were found between *APOE* genotypes and clinical markers, such as IOP and cup/disc ratio, in the PACG and PXG patients. These observations support the hypothesis proposed by previous studies that APOE may be involved in modulating RGC degeneration via an IOP-independent mechanism(s) [24,32,53].

There are several mechanisms through which APOE could potentially play a role in the pathogenesis of glaucoma. APOE is produced by astrocytes, neurons, retinal Müller cells, and macrophages [9,54], and variations in the binding properties of APOE isoforms across different cell types can have significant functional consequences at both the cellular and molecular levels [55]. Different APOE isoforms have been demonstrated to confer differing levels of risk associated with glaucoma. Animal experiments suggest that *APOE* gene deletion (*APOE*^−/−^) and the ε4 isoform may reduce the risk of RGC loss in glaucoma by inhibiting kainic acid receptor signaling, modulating microglial activation, and reducing galectin-3 expression [21,24]. Conversely, the presence of the ε3 isoform and overall *APOE* gene expression (*APOE*^+/+^) may increase the risk of RGC death in glaucoma by promoting microglial phenotypic changes and upregulating galectin-3 [22]. However, definitive evidence regarding the beneficial or detrimental impact of the ε2 allele in glaucoma is lacking in the current literature. Nonetheless, ε2 is reported to be associated with the highest APOE protein levels [56]. Therefore, it can be speculated that ε2 allele might increase the risk of PACG, as observed in our study, through any of the aforementioned mechanisms.

Moreover, the involvement of APOE in lipid metabolism, complement system regulation, neuroinflammation, blood–brain barrier integrity, oxidative stress, mitochondrial function, and angiogenesis contributing to Alzheimer’s or age-related macular degeneration pathogenesis [13,16,31] suggests multifaceted mechanisms through which APOE may contribute to the pathogenesis of PACG. Interestingly, new findings have revealed a role for APOE in regulating microRNA-controlled cellular signaling in cells of the immune system and vascular wall, suggesting a role of APOE in intercellular communication [57]. The presence of a similar mechanism in PACG cannot be ruled out. However, whether *APOE* genotypes influence these functions remains to be investigated.

Overall, the association of the *APOE* ε2 genotype with PACG requires a comprehensive understanding of the potential biological mechanisms related to aqueous humor dynamics, vascular factors, genetic interactions, comparative analysis with other ocular diseases, and consideration of population-specific factors. Further research into these aspects is essential for elucidating the role of *APOE* in PACG and its potential clinical implications. By contrast, the absence of this association in PXG may be attributed to differences in the underlying disease pathophysiology, genetic heterogeneity, environmental influences, or limitations in sample size and statistical power.

To conclude, our results show, for the first time, a positive association of *APOE* ε2-carriers in PACG, indicating the potential implication of ε2 in elevating the risk of PACG within the Saudi cohort. This observation suggests a possible role for *APOE* genetic variants in the pathogenesis of PACG, adding to our understanding of the genetic underpinnings of this complex ocular disorder among individuals of Saudi Arabian ancestry. However, the results require a cautious interpretation since this study is limited by sample size, especially in the subgroup analysis. Therefore, further validation incorporating larger population-based cohorts and molecular and functional studies are warranted to elucidate the underlying mechanisms and factors contributing to these observed associations.

## 4. Materials and Methods

### 4.1. Study Design, Ethics Approval, and Participant Characteristics

We conducted a retrospective and exploratory case-control study, sanctioned by the Institutional Review Board Ethics Committee at the College of Medicine of King Saud University as per the principles of the Declaration of Helsinki guidelines for human research. Participants were recruited at King Abdulaziz University Hospital in Riyadh, Saudi Arabia, as described elsewhere [58].

Briefly, PACG patients (*n* = 92) exhibited clinical signs of anatomically closed angles, elevated intraocular pressure (IOP) (≥21 mmHg), optic disc damage with a cup/disc ratio of at least 0.7, and visual field defects. PXG patients (*n* = 94) demonstrated the presence of exfoliation material along the pupil margins or anterior lens capsule, glaucomatous optic nerve damage, and elevated IOP. Exclusion criteria encompassed secondary glaucoma types, optic neuropathies not associated with glaucoma, corticosteroid use, ocular trauma, inadequate fundus visualization, or refusal to participate. Healthy age- and gender- matched controls (*n* = 251), aged ≥ 40 years, exhibited normal IOP, open angles on gonioscopy, healthy optic discs, and lacked a family history of glaucoma.

### 4.2. Genotyping rs429358 and rs7412 Polymorphisms in the APOE Gene

Peripheral EDTA blood samples were utilized for DNA extraction, followed by PCR amplification and Sanger sequencing to identify the rs429358 (T>C) and rs7412 (C>T) variants of the *APOE* gene, as previously described [28]. The primers used for PCR amplification, Sanger sequencing, and the cycling conditions are outlined in Table 6. In brief, DNA samples were PCR amplified, followed by purification using the QIAquick PCR Purification Kit (Qiagen, Hilden, Germany) and sequencing with M13 primers using the BigDye Terminator V3.1 Cycle Sequencing kit (Applied Biosystems, Foster City, CA, USA). Subsequently, sequencing analysis was performed on the ABI 3730 XL sequencer (Applied Biosystems), and nucleotide variations and *APOE* genotypes were determined using CLC Sequence Viewer 6.0 (Qiagen), in comparison to the *APOE* reference sequence (NG_007084.2).

### 4.3. Statistical Analysis

Statistical analyses were performed using SPSS version 25 (IBM Inc., Chicago, IL, USA) and SNPStats online software version 1.0. A significance threshold of *p* < 0.05 was applied, with Bonferroni’s correction for multiple testing (*p* = 0.05/2 = 0.025) where appropriate. Data normality was assessed using the Kolmogorov–Smirnov test. Continuous variables were analyzed by the Mann–Whitney U test and the Kruskal–Wallis test for two-group and three-group comparisons, respectively. The categorical variables and deviation from Hardy–Weinberg equilibrium were assessed using chi-square and Fisher’s exact tests, as applicable [30]. The impact of multiple factors, including age, sex, and genotypes, on the disease outcome was evaluated using binary logistic regression analysis.

## Figures and Tables

**Table 1:** Minor allele frequency distribution of *APOE* polymorphisms.

SNP ID	rs429358					rs7412				
Type	Minor Allele	MAF	OR (95% CI)	*p*	HWE *p*	Minor Allele	MAF	OR (95% CI)	*p*	HWE *p*
Controls	C	0.10	Reference	-	0.710	T	0.03	Reference	-	0.170
PACG	C	0.11	1.21 (0.70–2.1)	0.475	0.086	T	0.05	1.79 (0.76–4.21)	0.175	0.190
PXG	C	0.06	0.65 (0.33–1.24)	0.187	1.000	T	0.04	1.34 (0.53–3.40)	0.527	0.110

MAF—minor allele frequency, OR—odds ratio, 95% CI—95% confidence interval, HWE—Hardy–Weinberg Equilibrium, C—cytosine, T—thymine.

**Table 2:** Haplotype analysis of *APOE* polymorphisms according to *APOE* alleles and genotypes in primary angle-closure glaucoma (PACG).

*APOE*	Controls *n* (%)	PACG *n* (%)	OR (95% CI)	*p*
Alleles				
ε3	440 (87.6)	154 (83.7)	1.00	-
ε2	14 (2.8)	9 (4.9)	1.83 (0.78–4.32)	0.225
ε4	48 (9.6)	21 (11.4)	1.25 (0.72–2.15)	0.470
Genotypes ^a^				
ε3/ε3	199 (79.3)	66 (71.7)	1.00	-
ε2/ε2	1 (0.4)	1 (1.1)	3.01 (0.18–48.89)	0.999
ε2/ε3	4 (1.6)	7 (7.6)	5.27 (1.49–18.60)	0.009
ε2/ε4	8 (3.2)	0 (0)	-	0.205
ε3/ε4	38 (15.1)	15 (16.3)	1.19 (0.61–2.30)	0.730
ε4/ε4	1 (0.4)	3 (3.2)	9.04 (0.92–88.45)	0.053
ε3/ε3 vs. All	52 (20.7)	26 (28.2)	1.50 (0.87–2.60)	0.147
Carrier ^b^				
ε3/ε3	199 (81.9)	66 (71.7)	1.00	-
ε*2 ^c^	5 (2.0)	8 (8.7)	4.82 (1.52–15.26)	0.007
ε*4 ^d^	39 (16.0)	18 (19.5)	1.39 (0.74–2.60)	0.320

OR—odds ratio, 95% CI—95% confidence interval. ^a^ Overall Pearson chi-square = 16.36, df = 5, *p* = 0.006. ^b^ ε2/ε4 were excluded from either ε*2 or ε*4 group. ^c^ Includes ε2/ε2 and ε2/ε3. ^d^ Includes ε4/ε4 and ε3/ε4.

**Table 3:** Haplotype analysis of *APOE* polymorphisms according to *APOE* alleles and genotypes in pseudoexfoliation glaucoma (PXG).

*APOE*	Controls *n* (%)	PXG *n* (%)	OR (95% CI)	*p*
Alleles				
ε3	440 (87.6)	169 (89.9)	1.00	-
ε2	14 (2.8)	7 (3.7)	1.30 (0.51–3.28)	0.557
ε4	48 (9.6)	12 (6.4)	0.65 (0.33–1.25)	0.197
Genotypes ^a^				
ε3/ε3	199 (79.3)	77 (81.9)	1.00	-
ε2/ε2	1 (0.4)	1 (1.1)	2.58 (0.16–41.8)	0.999
ε2/ε3	4 (1.6)	4 (4.2)	2.80 (0.63–10.60)	0.230
ε2/ε4	8 (3.2)	1 (1.1)	0.32 (0.04–2.62)	0.452
ε3/ε4	38 (15.1)	11 (11.7)	0.75 (0.36–1.53)	0.488
ε4/ε4	1 (0.4)	0 (0)	-	0.999
ε3/ε3 vs. All	52 (20.7)	17 (18.0)	0.85 (0.46–1.55)	0.583
Carrier ^b^				
ε3/ε3	199 (81.9)	77 (82.8)	1.00	-
ε*2 ^c^	5 (2.0)	5 (5.4)	2.58 (0.72–9.17)	0.156
ε*4 ^d^	39 (16.0)	11 (11.8)	0.72 (0.35–1.49)	0.489

OR—odds ratio, 95% CI—95% confidence interval. ^a^ Overall Pearson chi-square = 4.80, df = 5, *p* = 0.441. ^b^ ε2/ε4 were excluded from either ε*2 or ε*4 carrier group. ^c^ Includes ε2/ε2 and ε2/ε3. ^d^ Includes ε4/ε4 and ε3/ε4.

**Table 4:** Binary logistic regression analysis of *APOE* genotypes effect in PACG and PXG.

Group Variables	B ^a^	SE	Wald	OR (95% CI)	*p*
PACG					
Age	0.023	0.017	1.812	1.02 (0.99–1.05)	0.178
Sex	−0.285	0.250	1.292	0.75 (0.46–1.23)	0.256
*APOE* genotypes			7.487		0.024
ε2-carriers ^b^	1.556	0.590	6.953	4.74 (1.49–15.06)	0.008
ε4-carriers ^c^	0.324	0.320	1.023	1.38 (0.74–2.59)	0.312
PXG					
Age	0.163	0.021	61.389	1.18 (1.130–1.22)	0.000
Sex	0.202	0.294	0.475	1.22 (0.69–2.17)	0.490
*APOE* genotypes			1.342		0.511
ε2-carriers ^b^	0.388	0.778	0.249	1.47 (0.32–6.77)	0.618
ε4-carriers ^c^	−0.438	0.436	1.010	0.64 (0.27–1.51)	0.315

OR—odds ratio, 95% CI—95% confidence interval. ^a^ B is the estimated coefficient, with standard error, SE. ^b^ Without ε4. ^c^ Without ε2.

**Table 5:** Haplotypes associated with *APOE* allele and their frequency distribution in different populations.

*APOE* Alleles	ε3	ε2	ε4
Haplotype	rs429358-T rs7412-C	rs429358-T rs7412-T	rs429358-C rs7412-C
Residue combination	112-Cys 158-Arg	112-Cys 158-Cys	112-Arg 158-Arg
Ethnicity	Allele frequency ^a^		
Europeans	0.640–0.900	0.044–0.120	0.052–0.310
Asians	0.620–0.870	0.020–0.140	0.071–0.240
Africans	0.536–0.850	0.031–0.116	0.085–0.407
Native Americans	0.720–0.911	0.0–0.014	0.089–0.280
Oceanians	0.486–0.740	0.0–0.145	0.260–0.368
Our study (Saudi Arabians) ^b^	0.876	0.028	0.096

^a^ Data from references [34,35]; ^b^ data from our controls.

**Table 6:** Primer used for PCR amplification and Sanger sequencing of *APOE* genotypes.

Primer Type	*APOE* Primer Sequences (5′–3′)	Thermal Cycling Conditions
Forward	^a^ GACCATGAAGGAGTTGAAGGCCTAC	Initial denaturation—95 °C for 15 min Cycling—95 °C—1 min, 59 °C—30 s, 72 °C—1 min for 35 cycles Final extension—72 °C—10 min
Reverse	^b^ GATGGCGCTGAGGCCGCGCT	

^a^ TGTAAAACGACGGCCAGT and ^b^ CAGGAAACAGCTATGACC M13 sequences were tagged at the 5′ end of the PCR primers and used for Sanger sequencing as described in Methods.

## Data Availability

The data supporting the conclusions of this article are all presented within the report.

## Supplementary Materials

The supporting information can be downloaded at: https://www.mdpi.com/article/10.3390/ijms25084571/s1.
