# Supplementary material for: APOE ε2-Carriers Are Associated with an Increased Risk of Primary Angle-Closure Glaucoma in Patients of Saudi Origin
Source: Int J Mol Sci. 2024 Apr 22;25(8):4571. doi: 10.3390/ijms25084571 (PMC11050284; doi:10.3390/ijms25084571)
Supplement: Supplementary file 1 [file ijms-25-04571-s001.zip › ijms-2963195-supplementary.pdf]

Figure S1: Association analysis of IOP and cup/disc ratio with rs429358 and rs7412 genotypes in primary angle-closure glaucoma patients in dominant model. * Outlier.

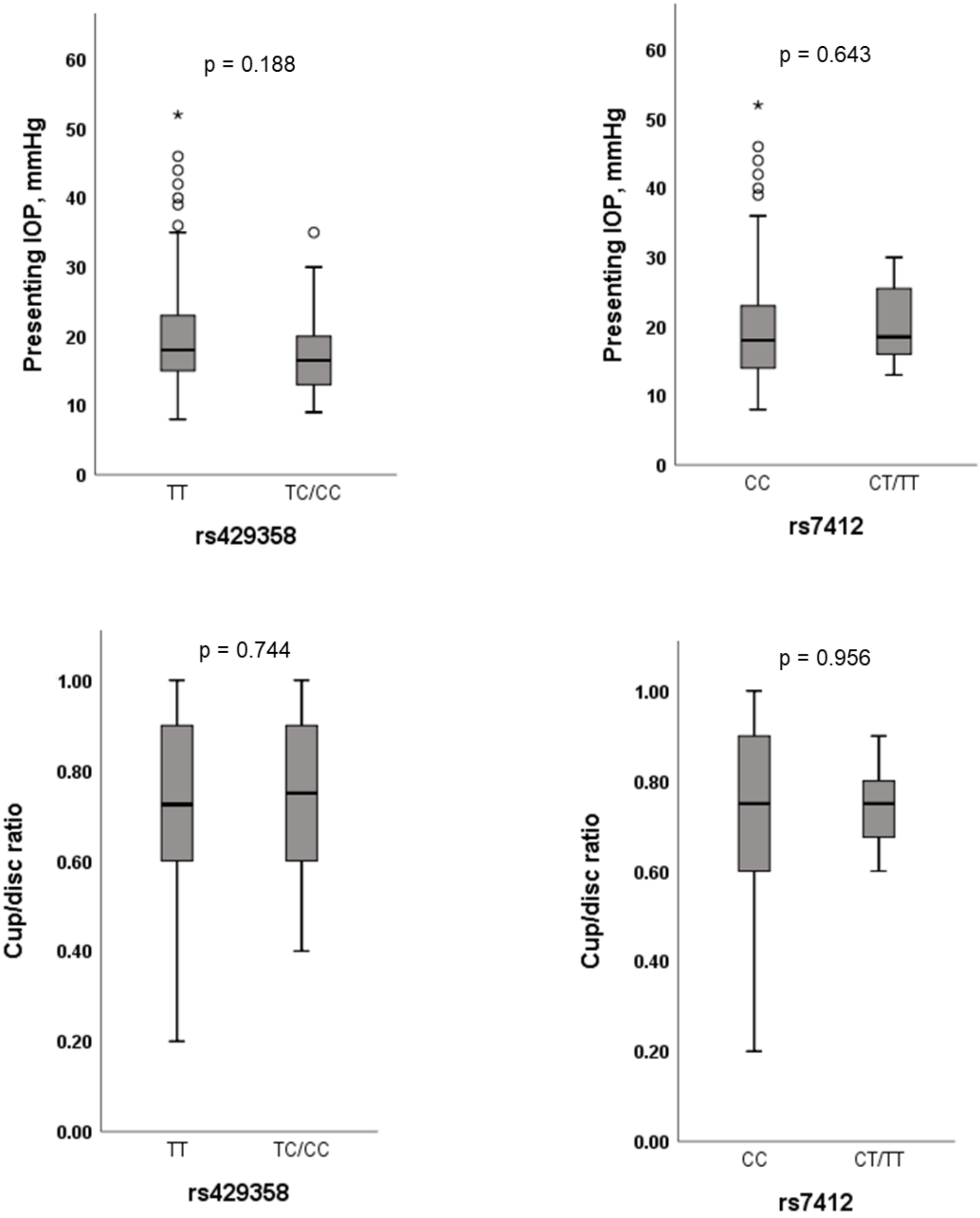

Figure S2: Association analysis of IOP and cup/disc ratio with rs429358 and rs7412 genotypes in pseudoexfoliation glaucoma patients in dominant model. * Outlier.

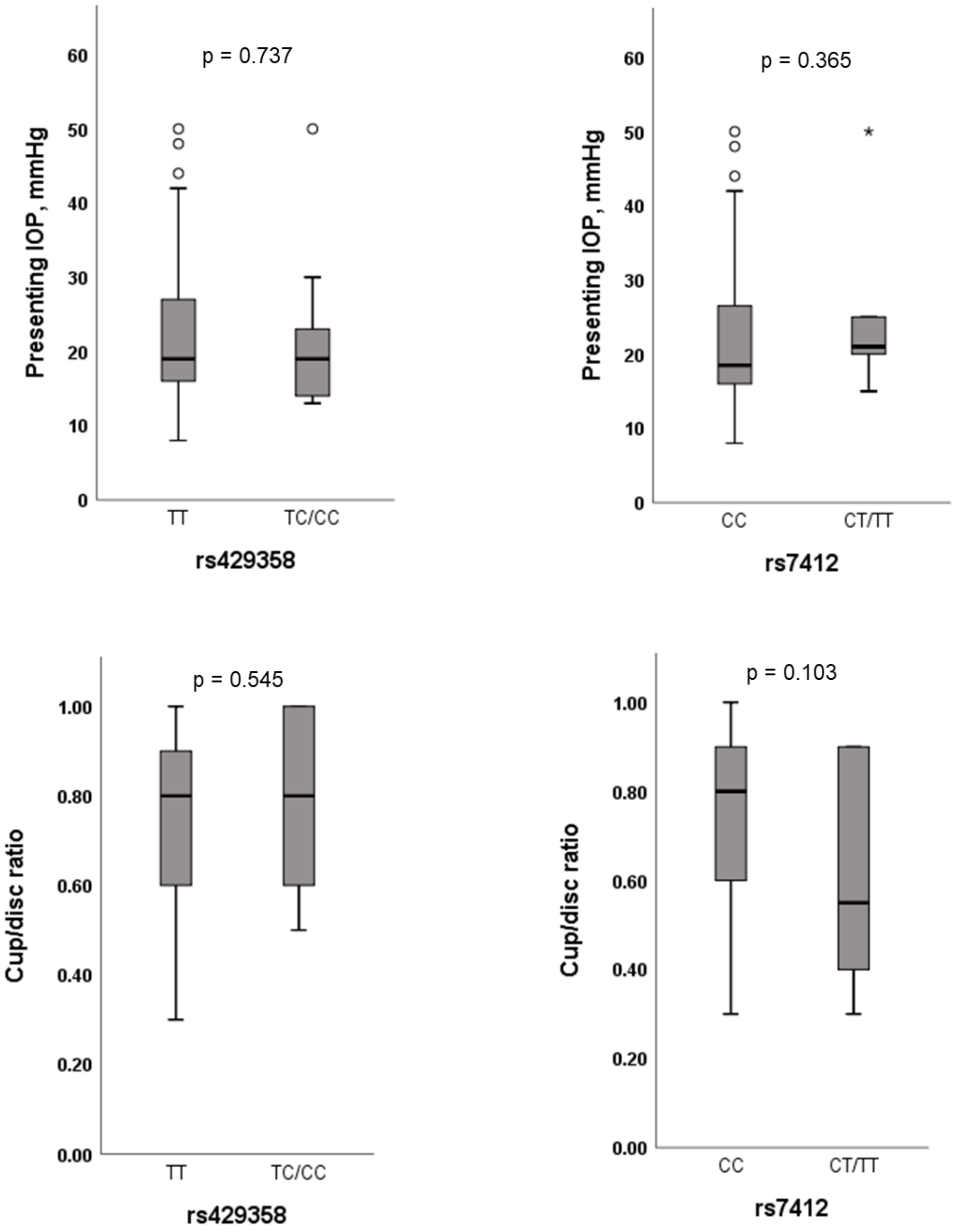

Table S1: Genotype analysis of rs429358 and rs7412 polymorphisms in *APOE* gene in primary angle-closure glaucoma (PACG)

SNP/Model	Genotype	Control	PACG	OR (95% CI)	*p*	AIC	BIC
rs429358							
Codominant	T/T	204 (81.3)	74 (80.4)	1.00	0.120	400.7	412.2
	T/C	46 (18.3)	15 (16.3)	0.90 (0.48-1.72)			
	C/C	1 (0.4)	3 (3.3)	7.15 (0.72-71.08)			
Dominant	T/T	204 (81.3)	74 (80.4)	1.00	0.860	402.9	410.5
	T/C-C/C	47 (18.7)	18 (19.6)	1.05 (0.57-1.93)			
Recessive	T/T-T/C	250 (99.6)	89 (96.7)	1.00	0.044§	398.8	406.5
	C/C	1 (0.4)	3 (3.3)	7.27 (0.73-72.12)			
rs7412							
Codominant	C/C	238 (94.8)	84 (91.3)	1.00			
	C/T	12 (4.8)	7 (7.6)	1.65 (0.63-4.34)	0.480	403.4	414.9
	T/T	1 (0.4)	1 (1.1)	2.83 (0.18-45.81)			
Dominant	C/C	238 (94.8)	84 (91.3)	1.00	0.240	401.5	409.2
	C/T-T/T	13 (5.2)	8 (8.7)	1.74 (0.70-4.35)			
Recessive	C/C-C/T	250 (99.6)	91 (98.9)	1.00	0.490	402.4	410.1
	T/T	1 (0.4)	1 (1.1)	2.75 (0.17-44.38)			

§ age and sex adjusted p-value was non-significant (p = 0.063)

OR – odds ratio, CI – confidence interval, AIC – Akaike information criterion, BIC – Bayesian information criterion

Table S2: Genotype analysis of rs429358 and rs7412 polymorphisms in *APOE* gene in pseudoexfoliation glaucoma (PXG)

SNP/Model	Genotype	Control	PXG	OR (95% CI)	$p^{§}$	AIC	BIC
rs429358							
Codominant	T/T	204 (81.3)	82 (87.2)	1.00	0.320	407.9	419.4
	T/C	46 (18.3)	12 (12.8)	0.65 (0.33-1.29)			
	C/C	1 (0.4)	0 (0)	0.00 (0.00-NA)			
Dominant	T/T	204 (81.3)	82 (87.2)	1.00	0.180	406.3	414
	T/C-C/C	47 (18.7)	12 (12.8)	0.64 (0.32-1.26)			
Recessive	T/T-T/C	250 (99.6)	94 (100)	1.00	0.420	407.5	415.2
	C/C	1 (0.4)	0 (0)	0.00 (0.00-NA)			
rs7412							
Codominant	C/C	238 (94.8)	88 (93.6)	1.00			
	C/T	12 (4.8)	5 (5.3)	1.13 (0.39-3.29)	0.770	409.6	421.1
	T/T	1 (0.4)	1 (1.1)	2.70 (0.17-43.71)			
Dominant	C/C	238 (94.8)	88 (93.6)	1.00	0.670	407.9	415.6
	C/T-T/T	13 (5.2)	6 (6.4)	1.25 (0.46-3.39)			
Recessive	C/C-C/T	250 (99.6)	93 (98.9)	1.00	0.490	407.7	415.3
	T/T	1 (0.4)	1 (1.1)	2.69 (0.17-43.42)			

§ age and sex adjusted p-value was non-significant

OR – odds ratio, CI – confidence interval, AIC – Akaike information criterion, BIC – Bayesian information criterion

Table S3: Binary logistic regression analysis in primary angle-closure glaucoma (PACG) and pseudoexfoliation glaucoma (PXG)

Group Variables	B[a]	SE	Wald	OR (95% CI)	*p*
PACG					
Age	0.016	0.017	0.969	1.02 (0.98-1.05)	0.325
Sex	-0.282	0.248	1.290	0.75 (0.46-.22)	0.256
rs429358			3.219	-	0.200
T/C	-0.152	0.334	0.207	0.86 (0.44-.65)	0.859
C/C	2.009	1.172	2.938	7.45 (0.75-74.16)	0.086
T/C+C/C	0.006	0.314	0.000	1.00 (0.55-1.86)	0.985
rs7412			1.758	-	0.415
C/T	0.587	0.503	1.365	1.80 (0.67-4.82)	0.243
C/C	0.925	1.429	0.419	2.52 (0.15-41.52)	0.517
C/T+C/C	0.555	0.474	1.370	1.74 (0.69-4.41)	0.242
PXG					
Age	0.168	0.021	64.086	1.18 (1.13-1.23)	0.000
Sex	0.195	0.293	0.441	1.21 (0.68-2.15)	0.507
rs429358			1.489		0.475
T/C	-0.520	0.426	1.489	0.59 (0.26-1.37)	0.222
C/C	-22.391	40192.970	0.000	0.00	1.000
T/C+C/C	-0.632	0.421	2.252	0.53 (0.23-1.21)	1.33
rs7412			0.243		0.886
C/T	-0.338	0.690	0.240	0.71 (0.18-2.76)	0.624
C/C	0.073	1.430	0.003	1.08 (0.07-17.74)	0.959
C/T+C/C	-0.211	0.620	0.115	0.81 (0.24-2.73)	0.734

OR – odds ratio, CI – confidence interval. [a] B is the estimated coefficient, with standard error, SE
